# Ecological Momentary Assessment Studies of Binge Eating in Non-Clinical Populations: A Scoping Review

**DOI:** 10.3390/nu18162655

**Published:** 2026-08-14

**Authors:** Jake Jeong, Giho Jeon, Kwangyeol Baek

**Affiliations:** 1 Department of Information Convergence Engineering, Pusan National University, Busan 50612, Republic of Korea; mekire@pusan.ac.kr (J.J.); ghjeon854@pusan.ac.kr (G.J.); 2 School of Biomedical Convergence Engineering, Pusan National University, Busan 50612, Republic of Korea

**Keywords:** binge eating, ecological momentary assessment, experience sampling method, non-clinical population, general population

## Abstract

**Background/Objectives**: Binge eating (BE)—eating an objectively large amount of food with a subjective loss of control—occurs broadly beyond formal eating disorder diagnoses. However, its real-time occurrence in the general population remains poorly understood due to reliance on retrospective, clinic-based self-reporting. Ecological momentary assessment (EMA) captures repeated, real-time reports of BE and may overcome these limitations. This scoping review mapped EMA studies of BE in non-clinical, general population samples, paying attention to population characteristics, methodological approaches, and key findings. **Methods**: This review followed PRISMA-ScR guidelines. PubMed and Scopus were searched for studies published between January 2016 and January 2026, with eligibility independently assessed by two reviewers. **Results**: Twenty-three studies met the inclusion criteria. Twenty studies (87%) comprised predominantly female samples, and most participants were in their twenties; men, middle-aged adults, and racially diverse populations were underrepresented. Regarding EMA methodology, BE was assessed either by measuring overeating and loss of control of eating separately (15 studies) or through direct, instructed self-report (8 studies); most studies used signal-contingent designs, with response rates generally being between 60% and 85%. Across studies, BE was most consistently linked to negative affect, weight stigma, and body dissatisfaction, while dietary restraint and food insecurity—a more recently emerging focus—were each examined in only one or two studies. **Conclusions**: This review maps the range of EMA methodologies used to assess BE, offering a methodological reference point for future studies. However, the existing evidence remains concentrated in young, female, and predominantly White samples, and further research in underrepresented populations is needed to clarify the broader determinants of BE in everyday life.

## 1. Introduction

Binge eating (BE) refers to consuming an objectively large amount of food within a discrete period accompanied by a subjective sense of loss of control (LOC) over eating [1]. This distinguishes BE from overeating (OE), which involves excessive intake without LOC, and from loss of control eating (LOCE), which may occur without objectively large amounts of food [2]. BE is a core symptom of binge eating disorder (BED) and bulimia nervosa (BN), but also occurs broadly among individuals who do not meet the full diagnostic criteria [3,4,5]. In the general population, BE has been associated with adverse mental and physical health outcomes, including depression, anxiety, obesity, and metabolic dysregulation, underscoring its public health relevance beyond clinical settings [3,5,6,7]. BED, for which BE is a core symptom, is the most common eating disorder presentation in the USA and globally, with community prevalence estimates in community samples ranging from approximately 0.85% to 2.6% [8,9]. BED is more common in women than in men, with women estimated to be about two to three times more likely to develop BED [9]. Individuals with BED show high rates of psychiatric comorbidity, particularly mood and anxiety disorders and substance use disorders [8,10], and are at increased risk for obesity, type 2 diabetes, hypertension, dyslipidemia, and metabolic syndrome, as well as impaired quality of life and psychosocial functioning [11].

Despite this relevance, empirical research on BE in non-clinical populations remains considerably limited, relative to clinical research. Studies have predominantly recruited treatment-seeking individuals or those with a formal eating disorder (ED) diagnosis, restricting the generalizability of findings to the broader population and limiting insight into the full subclinical spectrum of BE [12,13]. Furthermore, existing studies—including those conducted in community samples—have largely relied on retrospective self-report measures, typically asking participants to recall eating behavior over the preceding several weeks [14,15]. Given the episodic and time-limited nature of BE, such retrospective approaches are particularly susceptible to recall bias and are ill-suited to capturing the situational context and dynamic factors surrounding individual episodes [15,16]. Together, these limitations leave the real-world occurrence and momentary dynamics of BE in everyday life poorly understood.

Ecological momentary assessment (EMA), also known as the experience sampling method (ESM), is an ambulatory research methodology in which participants provide repeated self-reports in their natural environment across multiple time points throughout the day [16,17]. By capturing experiences in real time, EMA minimizes retrospective recall bias and preserves ecological validity, while enabling examination of intra-individual variability and temporal dynamics that cross-sectional or retrospective designs cannot detect. These features make EMA particularly well-suited for studying BE: it allows discrete episodes to be identified as they occur and facilitates examination of momentary affective, cognitive, and situational factors that precede or follow each episode—going beyond frequency counts to illuminate the processes underlying BE in daily life. Reflecting these advantages, a growing number of studies have employed EMA to examine BE. The most recent meta-analysis of BE-EMA research (k = 42 unique studies; N = 1745) confirmed this growth, yet also revealed that most samples comprised white females recruited from clinical or treatment-seeking contexts, with subthreshold and community-based populations underrepresented [18]. However, the existing EMA literature on BE has not yet been synthesized with sufficient attention to methodological variation. Individual studies differ in how BE is operationally defined—for example, whether episodes are identified through direct self-report or through the co-occurrence of objectively large food intake and loss of control—as well as in their EMA prompting schedules, event-contingent procedures, study duration, and response metrics. Studies also vary in the momentary affective, cognitive, behavioral, and contextual constructs assessed alongside BE. These differences make it difficult to determine how comparable findings are across studies and which methodological approaches are most commonly used in non-clinical populations. Although prior reviews and meta-analyses have summarized specific aspects of EMA research on BE, such as negative affect and hunger [15,18,19], a focused synthesis mapping operational definitions of BE, EMA protocols, and assessed constructs in non-clinical, general-population samples remains limited.

To address this gap, the present scoping review examined EMA/ESM research on BE in non-clinical, general-population samples. Specifically, we mapped the characteristics of study populations and synthesized the methodological approaches used across studies, including the operational definitions of BE episodes, EMA sampling and data-collection procedures, response metrics, and constructs assessed alongside BE. We also summarized the key variables and findings reported in relation to BE. On the basis of this synthesis, we identified methodological gaps and proposed directions for future research.

## 2. Materials and Methods

### 2.1. Search Strategy

This scoping review followed the PRISMA-ScR guidelines [20]. We systematically searched PubMed and Scopus for relevant articles published between January 2016 and January 2026.

The PubMed search utilized the following terms:

(“binge eating”[tiab] OR “binge-eating”[tiab]) AND (“ecological momentary assessment”[tiab] OR EMA[tiab] OR “experience sampling”[tiab] OR ESM[tiab]).

For Scopus, we applied the following:

TITLE-ABS-KEY(“binge eating” OR “binge-eating”) AND TITLE-ABS-KEY(“ecological momentary assessment” OR EMA OR “experience sampling” OR ESM).

### 2.2. Eligibility Criteria

We included and excluded studies according to predefined criteria focusing on EMA and ESM studies of binge-eating episodes. Studies were eligible if they (1) were published in English in peer-reviewed journals, (2) reported original data from human participants recruited from local communities or general populations (i.e., non-clinical settings), and (3) employed EMA or ESM to assess BE episodes as defined below.

We operationally defined BE according to DSM-5 as consuming an objectively large amount of food within a discrete period (OE) accompanied by a subjective sense of LOC, with simultaneous occurrences classified as BE episodes. Eligible studies assessed these episodes via EMA/ESM using one of two approaches: (A) explicit participant self-reports or logging of BE episodes (including timing and frequency) following instruction on BE definition in the DSM-5, or (B) event-contingent recording of eating episodes combined with EMA-derived, episode-level measures (e.g., OE and LOCE ratings) that enabled authors to classify episodes as BE using pre-specified operational criteria.

Studies were excluded if they were review articles, non-empirical publications, or conference abstracts lacking full text. We excluded studies involving clinical samples (e.g., individuals seeking treatment or diagnosed with EDs or other mental disorders) or those recruiting participants from hospitals or clinical settings. Studies lacking a direct EMA/ESM-based assessment of BE episodes were also excluded—specifically, studies in which no BE episodes were identified through (A) participant self-labeled reports or (B) author-defined classification of EMA eating episode logs using explicit a priori criteria.

Two authors independently conducted the screening process, which proceeded in two stages following duplicate removal: (1) title and abstract screening, and (2) full-text eligibility assessment. Search results from selected databases (PubMed, Scopus) were exported as CSV files, and duplicates were removed based on title, author, and year. Titles and abstracts were then evaluated for (A) recruitment from non-clinical samples and (B) use of EMA to assess BE. Potentially eligible full-text articles (excluding conference abstracts or other non-full-text publications) underwent detailed review for final eligibility. Discrepancies between reviewers were resolved through discussion during consensus meetings to reach agreement.

### 2.3. Data Extraction

After full-text eligibility review, data extraction was conducted using a pre-formatted Microsoft Excel spreadsheet. Extraction was carried out independently by two authors, who reconciled differences via discussion for content selected for summary tables. Information gathered from each study included: first author and publication year, country where research was performed, sample characteristics (size and demographics), EMA protocol specifics (questions used, BE criteria applied, EMA prompt frequency, study duration, and response rates), and primary results.

The individual article, rather than the independent participant sample, was treated as the unit of analysis because this review aimed to map methodological characteristics and findings across publications. Articles that appeared to draw on the same or overlapping samples were retained when they provided distinct methodological or substantive information relevant to the review. This decision was intended to preserve the breadth of the methodological literature, not to treat each article as an independent sample or an independent body of evidence. Because no pooled quantitative analyses were conducted, all counts should be interpreted at the article level rather than the independent-sample level.

### 2.4. Data Analysis and Synthesis

Data from the included articles were analyzed descriptively and synthesized narratively. The extracted information was organized into the following domains: study population characteristics, BE operationalization, EMA/ESM assessment procedures, sampling frequency and study duration, response or adherence metrics, and variables examined in relation to BE. Categorical characteristics were summarized using frequencies and percentages, whereas continuous characteristics were summarized using ranges, means, or medians when reported consistently across studies. Because the included articles varied substantially in study populations, BE definitions, EMA protocols, and outcome measures, the findings were not statistically pooled. Instead, articles were grouped according to shared methodological and substantive characteristics, and patterns and differences across studies were summarized using tables and narrative synthesis.

Where sufficient quantitative information was available, an exploratory Pearson correlation analysis was conducted to examine the association between daily EMA prompt frequency and response rate. This association was examined both with and without the study reporting the lowest response rate. No pooled effect estimates were calculated.

## 3. Results

### 3.1. Study Selection

**Figure 1:**
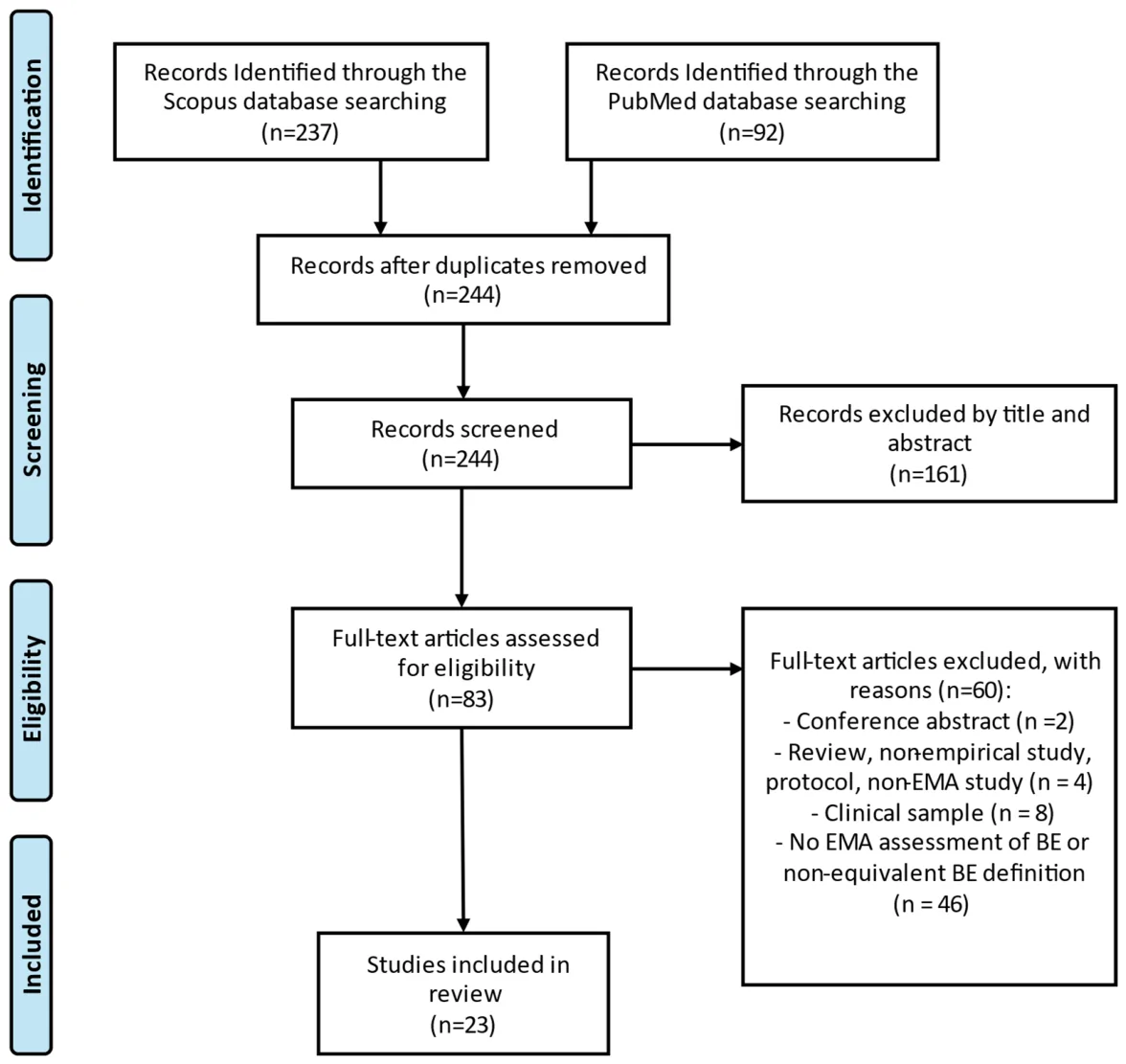
PRISMA flow chart of study inclusion.

Figure 1 presents the PRISMA flow diagram summarizing the overall study selection process. We initially identified 237 records through Scopus and 92 records through PubMed. After removing duplicates across databases, 244 unique records remained, of which 161 were excluded based on title and abstract screening. A total of 83 full-text articles were assessed for eligibility, and 23 studies met the inclusion criteria, were retained in the review, and are summarized in Table 1.

### 3.2. Sample Characteristics

#### 3.2.1. Sex/Gender, Age, Race and Ethnicity

Of the 23 included studies, 10 recruited exclusively female participants and 2 recruited exclusively male participants. The remaining 11 included mixed-gender samples, but women comprised the majority in most cases, with a mean proportion of 74.6%. Regarding age, the mean age was most commonly in the twenties (17 studies), followed by the forties (5 studies) and the teens (1 study); additionally, four studies specifically recruited college student samples [34,38,42,43], whereas Mason et al. [32] focused on middle-aged fathers (i.e., fathers with children aged 10–15 years). Furthermore, some studies included participants across a wide age range extending into the sixties [23,25,30], while others restricted the sample to participants in their thirties to forties [31,36,39]. In terms of study location, 16 studies were conducted in the United States, 4 in Estonia, and 1 each in Australia, Romania, and Canada. Most studies reported predominantly White samples (greater than 50%); however, Hazzard et al. [35] included a more racially diverse sample (21% White, 12% Black, 15% Hispanic, and 14% Asian).

#### 3.2.2. Sample Inclusion Criteria

Across the 23 included studies, 13 applied specific inclusion criteria, whereas the remaining 10 recruited general adult or university student samples without predefined eligibility restrictions. Only two studies used inclusion criteria related to disordered eating. Christensen Pacella et al. [37] recruited young women who reported at least one weekly episode of disordered eating behaviors (i.e., BE, purging, or restricting) during the previous three months. Nechita et al. [43], in contrast, included women with EDE-Q scores of 2.4 or higher. Seven studies applied weight-related inclusion criteria: Pearson et al. [24], Goldschmidt et al. [23], Smith et al. [25], and Anderson et al. [30] restricted their samples to adults with obesity, defined as a Body Mass Index (BMI) exceeding 30; Panza et al. [28,29] focused on sexually minoritized women with overweight or obesity; and Wetzel et al. [42] recruited female university students who perceived themselves as overweight/obese. Additionally, other studies employed more targeted criteria, with Romano et al. [36,41] recruiting women with body dissatisfaction, Hazzard et al. [35] focusing on young adults experiencing household food insecurity, and Moussaoui et al. [40] including adults who had engaged in self-injurious behavior within the past month.

### 3.3. EMA Methods

#### 3.3.1. Binge-Eating Assessment Items

Across studies, BE was operationalized in two main ways. The first approach, used in 15 studies, assessed objective OE and LOCE as separate components and classified BE episodes based on their co-occurrence. Within this approach, seven studies used dichotomous (yes/no) ratings for both OE and LOCE occurrence, and one study [38] elicited episode counts directly. The remaining seven used Likert-type scales with episodes counted above a pre-specified threshold. The second approach, used in eight studies, assessed BE episodes directly without separately coding OE and LOCE (e.g., “Did you actually binge eat since the last time you were signaled?”), following prior instruction provided to participants on the definition of BE. Within this approach, three studies used dichotomous (yes/no) occurrence ratings, two employed Likert-type response formats, and three asked participants to report the number of BE episodes within the specified EMA interval.

#### 3.3.2. EMA Frequency and Response Rate

Studies predominantly implemented one of three approaches: (1) signal-contingent designs using randomly timed prompts within predefined time windows, (2) combined signal-contingent and event-contingent designs, and (3) once-daily diary assessments, typically completed in the evening. Seventeen studies used signal-contingent designs, most of which delivered prompts at random times between approximately 09:00 and 22:00 with fixed or quasi-fixed intervals. The lowest prompting frequency was three prompts per day between morning and night [21,42], whereas the highest frequency was 10 prompts per day, delivered at least one hour apart between 09:00 and 23:00 [26]. Six studies combined signal-contingent and event-contingent sampling, with six signal-contingent prompts per day plus additional reports around focal events (e.g., before and after each eating episode). Two studies [36,41] implemented a daily diary design, sending brief surveys to participants’ mobile devices each evening.

**Figure 2:**
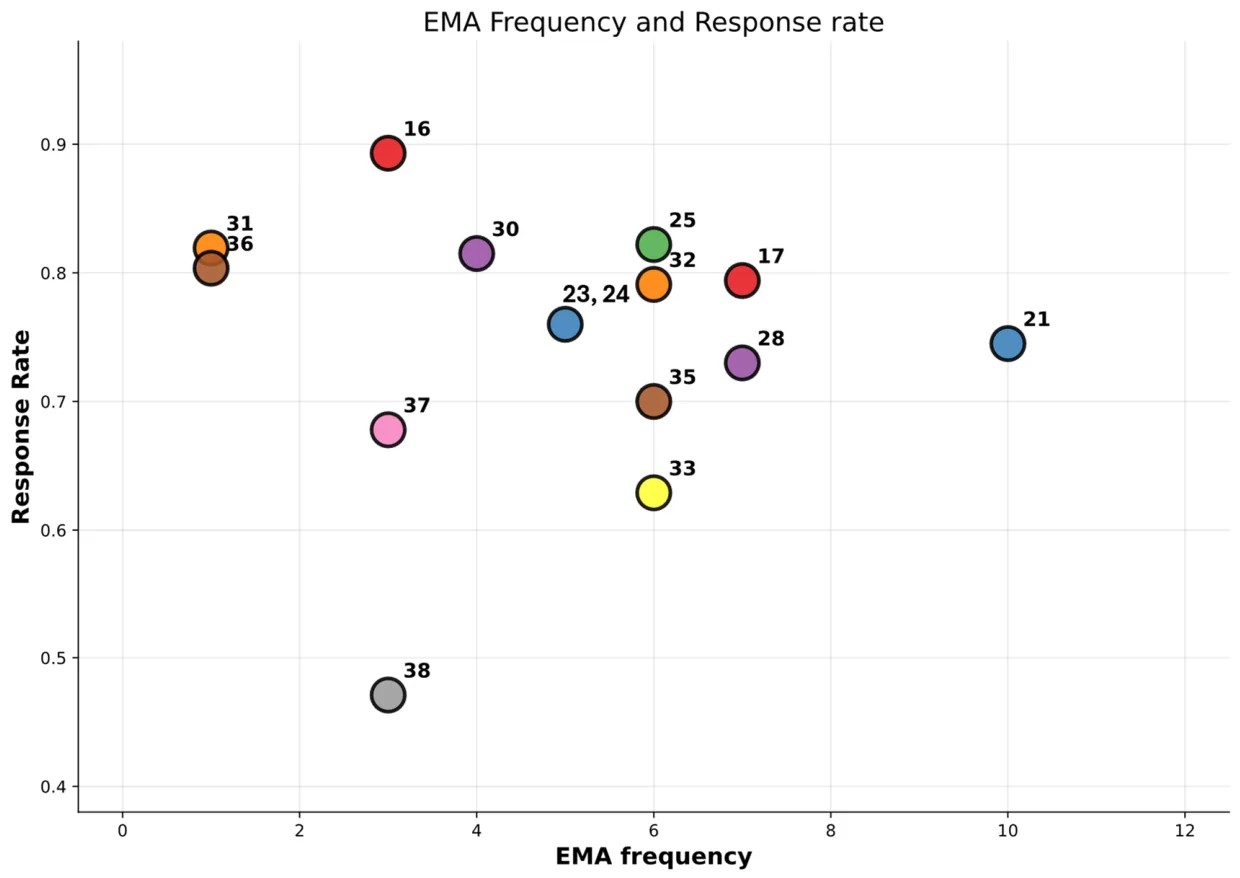
Association between EMA frequency and response rate.

Response metrics were reported heterogeneously. Sixteen of the 23 studies reported response rates, defined as the proportion of EMA prompts that received a response, whereas the remaining seven studies reported adherence rates, defined as the proportion of participants who completed the EMA study protocol. Among studies with response rates ≥ 80%, all but Anderson et al. [30] required no more than four responses per day. Most studies reported response rates between 60% and 85%, with only one study [43] reporting a notably lower response rate of 47.1%. As shown in Figure 2, response rate showed no significant association with daily EMA prompt frequency, regardless of whether Nechita et al. [43] was included (r = −0.061, *p* = 0.82) or excluded as an outlier (r = −0.35, *p* = 0.20). Adherence rates (the percentage of participants who were compliant with the study protocol) were reported in seven studies: three studies [23,24,25] reported an adherence rate of 92%, whereas the remaining four studies reported adherence rates of 71% [27], 79% [31], 76% [34], and 77% [39].

#### 3.3.3. EMA Duration

The duration of EMA monitoring ranged from 3 to 16 days. Thirteen studies collected EMA data for more than one week (≥8 days), whereas 10 studies employed monitoring periods of one week or less (≤7 days). The shortest protocols spanned three days [22,27,31,33], and the longest protocol extended to 16 days [40].

### 3.4. Major Findings (Variables Related to Binge Eating)

#### 3.4.1. Momentary Affect

Twelve studies examined associations between momentary affect and BE. Eight studies assessed momentary affect using EMA items adapted from the Positive and Negative Affect Schedule [44]. Seven studies [21,24,25,30,33,37,43] reported a positive association between momentary negative affect and BE (in terms of likelihood and/or severity), whereas Mason et al. [32] found that positive affect predicted greater BE severity and negative affect predicted lower BE severity in middle-aged fathers. Pearson et al. [24] reported a three-way interaction among negative affect, eating expectancies, and dietary restraint in predicting BE episodes. Anderson et al. [30] found that negative emotions, particularly disgust, mediated the association between feeling fat and BE. Additionally, Kukk et al. [27,31] reported that emotion regulation difficulties fully mediated and moderated the effect of negative affect on BE, while Christensen Pacella et al. [37] found that negative affect mediated the association between exposure to ED-salient content and BE.

#### 3.4.2. Body Image-Related Findings

Three studies focused on weight stigma in relation to BE. Panza et al. [29] reported that BE was associated with lifetime experiences of weight stigma and internalized weight bias. Romano et al. [36] showed that women’s daily weight stigma experiences predicted the likelihood of BE. In a subsequent study, Romano et al. [41] found that, following weight stigma experiences, Black women had higher odds of BE compared with White women. In a related domain, two studies examined body dissatisfaction as a moderator or predictor of BE: Fitzsimmons-Craft et al. [21] found that body dissatisfaction prospectively predicted an increased likelihood of BE episodes, whereas Tan et al. [26] reported a positive association between state body dissatisfaction and BE, with higher trait body image flexibility moderating this association. Furthermore, Panza et al. [28] found that BE was associated with state weight/shape concern, and Anderson et al. [30] reported that feeling fat was associated with BE. Nicoletta et al. [38] identified a positive association between appearance-focused self-concept and BE frequency.

#### 3.4.3. Dietary Restraint and Food Insecurity

Three studies examined associations between dietary restraint and BE: Pearson et al. [24] and Kukk et al. [31] found that dietary restraint was directly associated with BE, whereas Kukk et al. [27] reported that the urge to restrict fully mediated the association between negative affect and BE. In a related domain, Hazzard et al. [35] found that food security level was associated with BE severity, with food assistance, resource trade-off strategies, and self-efficacy moderating this association.

#### 3.4.4. Other Findings

Goldschmidt et al. [23] found that DSM-5 BE indicators were associated with self-reported BE episodes. Mason et al. [32] reported an association between BE severity and light physical activity. Fischer et al. [34] found that alcohol use and both positive and negative urgency traits were associated with BE. Moussaoui et al. [40] conducted a latent profile analysis of self-injurious urges and identified five profiles (sustained, muted, sudden-onset, volatile, and virtually absent), with individuals in the volatile profile showing the highest levels of engagement in BE compared with the other profiles. Finally, Wetzel et al. [42] reported that vigilant coping predicted BE.

## 4. Discussion

This scoping review mapped EMA/ESM studies examining BE in non-clinical, general population samples, with particular attention to study population characteristics, methodological approaches, and key variables and findings. This review extends prior work by focusing on EMA/ESM studies of BE in non-clinical, general-population samples and by systematically mapping how BE has been operationalized and assessed in real time across studies.

### 4.1. Study Population Characteristics

Twenty of the 23 included studies comprised predominantly female samples, whereas the remaining three reported male-majority samples. Notably, 10 studies recruited exclusively female samples, representing nearly half of the included studies. This imbalance likely reflects established epidemiological patterns, as the prevalence of ED and BE is consistently higher in women than in men [9,45,46,47]. EDs show some of the largest sex differences among psychiatric disorders, with women reported to be approximately four to nine times more likely to be affected [47,48,49], and BE is also more prevalent in women, even among non-clinical populations [50,51,52]. Sociocultural factors may further contribute, as women tend to report greater body image concerns and experience stronger appearance-related pressures, which promote internalization of thin ideals and engagement in restrictive eating behaviors. Given the higher prevalence of dieting among women [53,54], they may be more vulnerable to failures in dietary restraint, increasing the likelihood of BE episodes [55]. Together, these factors may explain both the focus on female samples and their higher participation rates in the literature. Nonetheless, because BE also occurs in men and may involve distinct risk profiles [22,33], further EMA research on male populations is needed.

In terms of age, 74% of studies reported mean participant ages in the twenties, with relatively few focusing on middle-aged or older adults. This pattern aligns with evidence that EDs most commonly develop during late adolescence and early adulthood [56,57]. Retrospective data estimate mean ages of onset at 19.7 years for bulimia nervosa and 25.4 years for BED [47]. However, recent longitudinal findings suggest a more nuanced age pattern [58]. ED prevalence appears to increase after age 45 in both men and women, with sex differences diminishing after age 50. While ED prevalence decreases with age in women, it remains relatively stable in men, alongside divergent trends in drive for thinness (decreasing in women, increasing in men). Additionally, some evidence suggests that men may experience later onset of ED and are less likely to seek professional help, often presenting with more severe comorbidities [59,60,61]. These findings highlight the need for further research on middle-aged populations, particularly among men.

Finally, most studies were conducted with predominantly White participants, and no studies specifically focused on Asian populations. This is consistent with the prior literature indicating that research on BE and BED has largely centered on White women [62]. However, given inconsistent findings regarding the role of race and ethnicity in ED [45,63], further research in more diverse populations is needed to improve generalizability.

### 4.2. EMA Methods

Across the included studies, BE was assessed either by measuring OE and LOCE separately and combining them, or by asking participants to report BE episodes directly after instruction of BE definition. The former, used in 15 of 23 studies, mirrors the DSM-5 conceptualization of BE as the conjunction of these two components, thereby allowing them to be analyzed both jointly and separately. The latter, used in the remaining eight studies, reduces the number of EMA prompts required per assessment, but its validity depends on participants’ accurate understanding and application of the instructed BE definition in the moment of report. Notably, both approaches appear to have been used productively across this body of literature, with studies under either method reporting associations between BE and various psychological, behavioral, and contextual variables.

EMA prompting schedules varied across studies. With the exception of two daily diary studies, all included studies used signal-contingent designs, and some also incorporated event-contingent sampling to collect prompts before and after eating episodes. Across studies, response rates were generally in the range of 60% to 85%, with only three studies reporting rates below 70% [38,42,43]. Meta-analyses of EMA compliance have suggested that a response rate of around 80% is appropriate [64,65], indicating that most studies in this review met or approached acceptable levels. Given prior findings that the within-day temporal patterns of BE and LOCE may differ among individuals with BED [66], signal-contingent designs alone may not fully capture temporal patterns of BE episodes, whereas incorporating event-contingent sampling can capture a broader range of relevant information.

### 4.3. Key Variables

Most studies examining momentary affect and BE reported a positive association between negative affect and BE. This finding is consistent with the Emotion Regulation Model, which proposes that BE serves as a response to negative emotion and as a strategy for avoiding or alleviating aversive affect [67]. Similarly, the two studies on emotion regulation consistently found that greater difficulties in emotion regulation were associated with BE [27,31]. These results align with prior evidence showing that individuals with BED tend to report stronger BE desire when they rely more heavily on maladaptive emotion regulation strategies [68]. However, because findings on emotion regulation strategies in individuals with BED have been mixed [69], additional research on their association with BE in non-clinical samples is needed.

In the body image-related studies, BE was associated with both weight stigma and body dissatisfaction. These findings are broadly consistent with the existing clinical literature, given that approximately 50% of individuals with BED report overvaluation of weight and shape [70]. However, the association between weight stigma and BE appears to vary somewhat across samples (e.g., specific racial groups or individuals seeking weight-loss treatment), suggesting the need for additional research in non-clinical samples [71]. Weight stigma and body dissatisfaction may contribute to psychological burden such as negative affect [71], which is itself associated with BE [67]. Further studies are needed to clarify the temporal ordering of these relationships.

Dietary restraint was associated with BE [24,31], in line with the well-established link between restrained eating and BE. Food insecurity, in contrast, has only recently emerged as a topic of interest in BE research [72]. Within the present review, Hazzard et al. [35] was the only study to examine this association, finding that food security level was associated with BE symptoms. Given that BE episodes often involve consumption of highly palatable foods, further research is needed to clarify how food insecurity contributes to BE risk in non-clinical populations.

### 4.4. Strengths and Limitations

This study has several limitations. First, we only searched PubMed and Scopus and did not include psychology-specific databases such as PsycINFO. These two databases offer broad, complementary coverage of the relevant health-sciences literature, but studies only indexed elsewhere may have been missed. Eligibility was also restricted to peer-reviewed, full-text articles published in English within the past 10 years, so relevant work in other languages, in the gray literature, or outside this window may have been overlooked. Second, our focus on non-clinical samples excluded clinical and treatment-seeking populations by design. Moreover, several included samples were at elevated risk (e.g., obesity, food insecurity, or elevated eating-disorder symptoms), so the boundary of “non-clinical” varied across studies. Third, some findings (Section 3.4.4) were not discussed in depth, given their heterogeneity and few supporting studies. Finally, some articles may have drawn on overlapping participant samples, although the extent of overlap could not be fully verified from the published reports. Because the article was treated as the unit of analysis, the 23 included articles should not be interpreted as representing 23 independent samples or independent bodies of evidence. Articles reporting distinct methodological or substantive findings were retained to preserve the breadth of the literature; however, this approach may overstate the apparent size of the evidence base if article counts are interpreted as counts of independent studies. Future reviews should identify and link publications arising from the same participant samples and report both article-level and independent-sample-level counts whenever possible.

Nevertheless, this review has several strengths that distinguish it from prior work. First, it deliberately focuses on non-clinical, community-based samples—populations underrepresented in the BE-EMA literature, which has centered largely on clinical and treatment-seeking groups—thereby mapping evidence on the often-overlooked subclinical spectrum of binge eating. Second, it provides a structured synthesis of how binge eating has been operationally defined in real time across studies, an aspect rarely examined systematically in prior reviews. Third, by cataloging these definitions alongside EMA protocols and assessed constructs, the review makes the methodological heterogeneity of the field explicit and locates where evidence is currently lacking. Finally, it followed a reproducible search strategy and adhered to established methodological recommendations for scoping reviews.

## 5. Conclusions

This scoping review mapped 23 articles examining BE using EMA/ESM in non-clinical, general-population samples. Across studies, BE was assessed either by combining separate measures of OE and LOCE or through direct participant reports, but substantial variation was observed in operational definitions, sampling procedures, monitoring duration, and response metrics.

Future EMA studies should recruit more diverse samples, particularly men, middle-aged and older adults, and participants from underrepresented racial and ethnic groups. Researchers should also prespecify and transparently report BE classification criteria, including how OE and LOC are assessed and combined, and should use clearly described and sufficiently comparable EMA protocols. Consistent reporting of prompting schedules, event-contingent assessments, monitoring duration, and response or adherence rates would improve comparability across studies. Finally, longitudinal and event-sensitive designs are needed to clarify the temporal relationships among affect, body-image concerns, dietary restraint, and BE in everyday life.

## Figures and Tables

**Table 1:** Summary of the included studies, EMA protocols, and key findings.

Study (Year, Nation)	Participants (Female %)	Age (Mean ± SD)	EMA Items: BE	EMA Items: Others	EMA Frequency	Study Duration	Response Rate	Main Finding
Fitzsimmons-Craft et al. (2016, USA) [21]	232 women university students (100%)	18.7 ± 1.0	1 item, yes/no (BE, instructed)	Disordered eating (behavior, thought/urge), Social comparison (body, eating, exercise), Body dissatisfaction, Negative affect	3/day	14 days	89.3%	(1) Body dissatisfaction and negative affect prospectively predicted BE urge. (2) Eating comparison and body dissatisfaction prospectively predicted likelihood of BE.
Kukk et al. (2017, Estonia) [22]	158 adults (61.4%)	22.4 ± 5.0	2 items, yes/no (OE 1, LOCE 1)	Momentary feeling	7/day	3 days	79.4%	(1) Negative emotion fluctuations predicted BE. (2) Gender differences in the predictors of BE (women: emotion regulation difficulties; men: trait impulsivity).
Goldschmidt et al. (2018, USA) [23]	50 adults with obesity (84%)	43.0 ± 11.9	2 items, Likert (OE 1, LOCE 1)	Eating episode (duration/satiety/feeling)	6/day +event based	14 days	-	All DSM-5 binge-eating indicators except eating rapidly were associated with self-reported BE episodes.
Pearson et al. (2018, USA) [24]	50 adults with obesity (84%)	43.0 ± 11.9	2 items, Likert (OE 1, LOCE 1)	Dietary restraint, Negative affect, Eating expectation	6/day +event based	14 days	-	(1) Dietary restraint predicted likelihood of BE. (2) Three-way interaction (momentary negative affect, eating expectancies, and dietary restraint) predicted BE episodes.
Smith et al. (2018, USA) [25]	50 adults with obesity (84%)	43.0 ± 11.9	2 items, Likert (OE 1, LOCE 1)	Restraint, Affect	6/day +event based	14 days	-	Negative affect (state & trait) predicted BE episodes.
Tan et al. (2019, Australia) [26]	147 women (100%)	22.2 ± 5.0	2 items, yes/no (OE 1, LOCE 1)	Body dissatisfaction, Dieting, Unhealthy eating, Drive for thinness, Appearance comparison	10/day	7 days	74.5%	(1) Trait body image flexibility predicted lower BE frequency. (2) BE predicted state body dissatisfaction (Moderator: trait body image flexibility).
Kukk et al. (2020, Estonia) [27]	104 men (0%)	27.3 ± 8.0	2 items, yes/no (OE 1, LOCE 1)	Urge to restrict, Negative affect	7/day	3–7 days	-	Emotion regulation difficulties and the urge to restrict mediated the effect of negative affect on BE.
Panza et al. (2021, USA) [28]	55 Sexually minority women with overweight/obesity (100%)	25.0 ± 9.0	2 items, yes/no (OE 1, LOCE 1)	State weight/shape concern	5/day	5 days	76.0%	(1) Trait eating concern was associated with BE. (2) State weight/shape concern were associated with concurrent/subsequent BE.
Panza et al. (2021, USA) [29]	55 Sexually minority women with overweight/obesity (100%)	25.0 ± 9.0	2 items, yes/no (OE 1, LOCE 1)	-	5/day	5 days	76.0%	(1) Internalized homophobia, (2) Lifetime weight stigma events, and (3) Internalized weight bias was associated with BE episode.
Anderson (2022, USA) [30]	50 adults with obesity (84%)	43.0 ± 11.9	5 items, Likert (OE 1, LOCE 4)	Feeling fat, Negative affect	6/day +event based	14 days	82.2%	(1) Feeling fat was associated with BE. (2) Negative emotions (particularly disgust) mediated the effect of feeling fat on BE.
Kukk (2022, Estonia) [31]	96 women (100%)	21.5 ± 6.7	2 items, yes/no (OE 1, LOCE 1)	Restraint, Negative affect	7/day	3 days	-	(1) Emotion regulation difficulties moderate the effect of negative affect on BE. (2) Dietary restraint predicted BE.
Mason et al. (2022, USA) [32]	23 middle-aged fathers (0%)	46.5 ± 6.7	2 items, Likert (OE 1, LOCE 1)	Affect, Food intake, Activity (light activity, moderate-to-vigorous physical activity, sedentary time)	4–8/day (weekday 4, weekend 8)	8 days	72.0%	(1) Positive and negative affect predicted BE severity. (2) Sweets and fast-food intake predicted BE severity. (3) Light activity predicted BE severity.
Sultson et al. (2022, Estonia) [33]	181 adults (48.1%)	24.3 ± 6.7	2 items, yes/no (OE 1, LOCE 1)	Affect, Situation/social context (place, social context)	7/day	3 days	73.0%	(1) In women, negative affect was highest before BE, followed by OE compared to no OE/BE. (2) In men, positive affect was significantly higher before OE compared to BE and no OE/BE. (3) In men, BE was more prevalent when with others, especially at home (vs. alone).
Fischer et al. (2023, USA) [34]	106 university students (76.4%)	20.8 ± 3.6	1 item, yes/no (BE, instructed)	Drinking, Motives (eating and drinking), Affect	6/day + event based	14 days	-	(1) Alcohol use predicted later BE. (2) Elevated odds of BE after drinking were associated with positive urgency and negative urgency traits.
Hazzard et al. (2023, USA) [35]	75 young adults under household food insecurity (72%)	25.3 ± 1.8	4 items, Likert (OE 2, LOCE 2)	Food security	4/day	14 days	81.5%	(1) Instances of food security predicted subsequent BE symptoms. (2) Food assistance, resource trade-off coping strategies, food security-related self-efficacy moderated this association.
Romano et al. (2023, USA) [36]	198 young adults with body dissatisfaction (87.9%)	21.1 ± 3.8	1 item, No. of daily BE (instructed)	Disordered/Intuitive eating behaviors, Body dissatisfaction, Weight Stigma	1/day	14 days	81.93%	Women’s daily weight stigma experience predicted likelihood of BE (within-person).
Christensen Pacella et al. (2024, USA) [37]	173 women with disordered eating behaviors (100%)	20.2 ± 1.70	1 item, No. of BE episode (instructed)	Disordered eating behaviors, Instagram use, Affect, ED-salient content exposure	6/day	7 days	79.1%	Negative affect partially mediated the effects of ED-salient content exposure on subsequent BE and restrictive eating.
Nicoletta et al. (2024, Canada) [38]	63 female university students (100%)	22.1 ± 4.9	2 items, No. of episode (OE 1, LOCE 1)	Appearance-focused self-concept, Dietary restraint	6/day	14 days	62.9%	Appearance-focused self-concept (both morning levels and daytime increases) predicted greater same-day BE frequency.
Smith et al. (2024, USA) [39]	24 adults (66.7%)	22.3 ± 4.0	2 items, Likert (OE 1, LOCE 1)	Craving, Dietary restraint, Affect	4/day	10 days	-	Higher positive affect instability was associated with higher ratings of BE symptoms (within-person).
Moussaoui et al. (2025, USA) [40]	124 adults with self-injurious behaviors (71.9%)	22.8 ± 9.7	1 item, yes/no (BE, instructed)	Self-injurious urges, Self-injurious behaviors	6/day + event based	16 days	70.0%	Latent profile analysis identified five self-injurious urge profiles; BE was most frequent in the volatile profile.
Romano et al. (2025, USA) [41]	144 women with body dissatisfaction (100%)	28.8 ± 7.6	1 item, No. of daily BE (instructed)	Daily weight stigma experience, Disordered eating behaviors	1/day	14 days	80.4%	When experiencing weight stigma, Black women showed higher odds of BE compared with White women.
Wetzel et al. (2025, USA) [42]	130 female university students with self-perceived overweight/obesity (100%)	20.6 ± 5.1	1 item, Likert (BE, instructed)	Weight stigma, Vigilance, Eating to cope, Restrictive eating	3/day	7 days	67.8%	Vigilant coping predicted BE (between-person).
Nechita et al. (2026, Rumania) [43]	93 women with ED symptoms (100%)	28.6 ± 8.6	1 item, Likert (BE, instructed)	Disturbed eating behavior, Negative affect, Self-efficacy, General shame (body- and eating-related)	5/day	7 days	47.1%	(1) High-shame episodes predicted subsequent BE severity. (2) Mobile self-compassion intervention prevented post-shame increases in BE.

Abbreviations: BE, binge eating; DSM, Diagnostic and Statistical Manual of Mental Disorders, 5th edition; ED, eating disorder; EMA, ecological momentary assessment; LOCE, loss-of-control eating; OE, overeating; SD, standard deviation.

## Data Availability

No new data were created or analyzed in this study. Data sharing is not applicable to this article.

## Abbreviations

The following abbreviations are used in this manuscript:

BE	Binge eating
BED	Binge eating disorder
BMI	Body mass index
BN	Bulimia nervosa
DSM-5	Diagnostic and Statistical Manual of Mental Disorders, Fifth Edition
ED	Eating disorder
EDE-Q	Eating Disorder Examination Questionnaire
EMA	Ecological momentary assessment
ESM	Experience sampling method
LOC	Loss of control
LOCE	Loss of control eating
OE	Overeating
PRISMA-ScR	Preferred Reporting Items for Systematic Reviews and Meta-Analyses Extension for Scoping Reviews
SD	Standard deviation
USA	United States of America

## References

[1] American Psychiatric Association (2013). Diagnostic and Statistical Manual of Mental Disorders: DSM-5.

[2] Goldschmidt A.B. (2017). Are loss of control while eating and overeating valid constructs? A critical review of the literature. Obes. Rev..

[3] Appolinario J.C., Sichieri R., Lopes C.S., Moraes C.E., da Veiga G.V., Freitas S., Nunes M.A., Wang Y.-P., Hay P. (2022). Correlates and impact of DSM-5 binge eating disorder, bulimia nervosa and recurrent binge eating: A representative population survey in a middle-income country. Soc. Psychiatry Psychiatr. Epidemiol..

[4] Mitchison D., Touyz S., González-Chica D.A., Stocks N., Hay P. (2017). How abnormal is binge eating? 18-Year time trends in population prevalence and burden. Acta Psychiatr. Scand..

[5] Derks I.P., Harris H.A., Staats S., Gaillard R., Dieleman G.C., Llewellyn C.H., Swanson S.A., Jansen P.W. (2022). Subclinical binge eating symptoms in early adolescence and its preceding and concurrent factors: A population-based study. J. Eat. Disord..

[6] Baek K. (2025). The Role of Binge Eating in a Sequential Mediation Model of Stress, Emotional Eating, and BMI. Nutrients.

[7] Pruccoli J., Mack I., Klos B., Schild S., Stengel A., Zipfel S., Giel K.E., Schag K. (2023). Mental health variables impact weight loss, especially in patients with obesity and binge eating: A mediation model on the role of eating disorder pathology. Nutrients.

[8] Kessler R.C., Berglund P.A., Chiu W.T., Deitz A.C., Hudson J.I., Shahly V., Aguilar-Gaxiola S., Alonso J., Angermeyer M.C., Benjet C. (2013). The prevalence and correlates of binge eating disorder in the World Health Organization World Mental Health Surveys. Biol. Psychiatry.

[9] Udo T., Grilo C.M. (2018). Prevalence and correlates of DSM-5–defined eating disorders in a nationally representative sample of US adults. Biol. Psychiatry.

[10] Bogusz K., Kopera M., Jakubczyk A., Trucco E.M., Kucharska K., Walenda A., Wojnar M. (2021). Prevalence of alcohol use disorder among individuals who binge eat: A systematic review and meta-analysis. Addiction.

[11] Hudson J.I., Lalonde J.K., Coit C.E., Tsuang M.T., McElroy S.L., Crow S.J., Bulik C.M., Hudson M.S., Yanovski J.A., Rosenthal N.R. (2010). Longitudinal study of the diagnosis of components of the metabolic syndrome in individuals with binge-eating disorder. Am. J. Clin. Nutr..

[12] Davis C. (2013). From passive overeating to “food addiction”: A spectrum of compulsion and severity. Int. Sch. Res. Not..

[13] Striegel-Moore R.H., Dohm F.-A., Solomon E., Fairburn C.G., Pike K.M., Wilfley D.E. (2000). Subthreshold binge eating disorder. Int. J. Eat. Disord..

[14] Berg K.C., Peterson C.B., Frazier P., Crow S.J. (2012). Psychometric evaluation of the eating disorder examination and eating disorder examination-questionnaire: A systematic review of the literature. Int. J. Eat. Disord..

[15] Haedt-Matt A.A., Keel P.K. (2011). Revisiting the affect regulation model of binge eating: A meta-analysis of studies using ecological momentary assessment. Psychol. Bull..

[16] Schaefer L.M., Engel S.G., Wonderlich S.A. (2020). Ecological momentary assessment in eating disorders research: Recent findings and promising new directions. Curr. Opin. Psychiatry.

[17] Shiffman S., Stone A.A., Hufford M.R. (2008). Ecological momentary assessment. Annu. Rev. Clin. Psychol..

[18] Borm I.M., Hartmann S., Barnow S., Pruessner L. (2025). Binge eating as emotion regulation? A meta-analysis of ecological momentary assessment studies. Clin. Psychol. Rev..

[19] Haedt-Matt A.A., Keel P.K. (2011). Hunger and binge eating: A meta-analysis of studies using ecological momentary assessment. Int. J. Eat. Disord..

[20] Tricco A.C., Lillie E., Zarin W., O’Brien K.K., Colquhoun H., Levac D., Moher D., Peters M.D., Horsley T., Weeks L. (2018). PRISMA extension for scoping reviews (PRISMA-ScR): Checklist and explanation. Ann. Intern. Med..

[21] Fitzsimmons-Craft E.E., Ciao A.C., Accurso E.C. (2016). A naturalistic examination of social comparisons and disordered eating thoughts, urges, and behaviors in college women. Int. J. Eat. Disord..

[22] Kukk K., Akkermann K. (2017). Fluctuations in negative emotions predict binge eating both in women and men: An experience sampling study. Eat. Disord..

[23] Goldschmidt A.B., Crosby R.D., Cao L., Wonderlich S.A., Mitchell J.E., Engel S.G., Peterson C.B. (2018). A preliminary study of momentary, naturalistic indicators of binge-eating episodes in adults with obesity. Int. J. Eat. Disord..

[24] Pearson C.M., Mason T.B., Cao L., Goldschmidt A.B., Lavender J.M., Crosby R.D., Crow S.J., Engel S.G., Wonderlich S.A., Peterson C.B. (2018). A test of a state-based, self-control theory of binge eating in adults with obesity. Eat. Disord..

[25] Smith K.E., Mason T.B., Crosby R.D., Engel S.G., Crow S.J., Wonderlich S.A., Peterson C.B. (2018). State and trait positive and negative affectivity in relation to restraint intention and binge eating among adults with obesity. Appetite.

[26] Tan W., Holt N., Krug I., Ling M., Klettke B., Linardon J., Baxter K., Hemmings S., Howard D., Hughes E. (2019). Trait body image flexibility as a predictor of body image states in everyday life of young Australian women. Body Image.

[27] Kukk K., Akkermann K. (2020). Emotion regulation difficulties and dietary restraint independently predict binge eating among men. Eat. Weight Disord.-Stud. Anorex. Bulim. Obes..

[28] Panza E., Olson K., Selby E.A., Wing R.R. (2021). State versus trait weight, shape, and eating concerns: Disentangling influence on eating behaviors among sexual minority women. Body Image.

[29] Panza E., Fehling K.B., Pantalone D.W., Dodson S., Selby E.A. (2021). Multiply marginalized: Linking minority stress due to sexual orientation, gender, and weight to dysregulated eating among sexual minority women of higher body weight. Psychol. Sex. Orientat. Gend. Divers..

[30] Anderson L.M., Hall L.M., Crosby R.D., Crow S.J., Berg K.C., Durkin N.E., Engel S.G., Peterson C.B. (2022). “Feeling fat,” disgust, guilt, and shame: Preliminary evaluation of a mediation model of binge-eating in adults with higher-weight bodies. Body Image.

[31] Kukk K., Akkermann K. (2022). The interplay between binge eating risk factors: Toward an integrated model. J. Health Psychol..

[32] Mason T.B., Do B., Chu D., Belcher B.R., Dunton G.F., Lopez N.V. (2022). Associations among affect, diet, and activity and binge-eating severity using ecological momentary assessment in a non-clinical sample of middle-aged fathers. Eat. Weight Disord.-Stud. Anorex. Bulim. Obes..

[33] Sultson H., Kreegipuu K., Akkermann K. (2022). Exploring the role of momentary positive and negative affect in overeating and binge eating: Evidence for different associations among men and women. Appetite.

[34] Fischer S., Wonderlich J., Miller L.A., Breithaupt L., Frietchen R., Cao L., Nelson J.D., Izquierdo A. (2023). The association of alcohol use and positive and negative urgency to same day objective binge eating in emerging adults. Front. Psychol..

[35] Hazzard V.M., Loth K.A., Crosby R.D., Wonderlich S.A., Engel S.G., Larson N., Neumark-Sztainer D. (2023). Relative food abundance predicts greater binge-eating symptoms in subsequent hours among young adults experiencing food insecurity: Support for the “feast-or-famine” cycle hypothesis from an ecological momentary assessment study. Appetite.

[36] Romano K.A., Heron K.E. (2023). Daily weight stigma experiences, and disordered and intuitive eating behaviors among young adults with body dissatisfaction. Int. J. Eat. Disord..

[37] Christensen Pacella K.A., Forbush K.T., Chen Y., Nation M.B., Cushing C.C., Swinburne Romine R.E. (2024). Negative affect as a mediator between exposure to fitspiration and thinspiration and disordered eating behaviors: An ecological momentary assessment study. Int. J. Eat. Disord..

[38] Nicoletta J., Mosco R., Enouy S., Tabri N. (2024). Momentary appearance focused self-concept is associated with dietary restraint and binge eating in female university students: An experience sampling study. Int. J. Eat. Disord..

[39] Smith A., Page K.A., Smith K.E. (2024). Associations between affect dynamics and eating regulation in daily life: A preliminary ecological momentary assessment study. Cogn. Emot..

[40] Moussaoui J.R., Smith A.R., Velkoff E.A. (2025). Latent subtypes of self-injurious urges among adults engaging in disordered eating and non-suicidal self-injury. Suicide Life-Threat. Behav..

[41] Romano K.A., Panza E., Peterson C.B., Hooper L., Mason T.B. (2025). (Mis) matches in daily weight stigma perpetrators’ and targets’ genders and races relative to targets’ daily disordered eating behaviors: Examining differences between Black and White women. J. Acad. Nutr. Diet..

[42] Wetzel K.E., Himmelstein M.S., Ciesla J.A. (2025). Bracing for impact: An intensive longitudinal investigation of weight stigma, vigilant coping, and maladaptive eating. Soc. Sci. Med..

[43] Nechita D.-M., Matu S.-A. (2026). Smartphone-delivered ecological momentary interventions for disordered eating following intense shame experiences. Appetite.

[44] Watson D., Clark L.A., Tellegen A. (1988). Development and validation of brief measures of positive and negative affect: The PANAS scales. J. Personal. Soc. Psychol..

[45] Wick M.R., Fitzgerald E.H., Keel P.K. (2020). Epidemiology of binge eating. Binge Eating: A Transdiagnostic Psychopathology.

[46] Breton É., Juster R.P., Booij L. (2023). Gender and sex in eating disorders: A narrative review of the current state of knowledge, research gaps, and recommendations. Brain Behav..

[47] Hudson J.I., Hiripi E., Pope H.G., Kessler R.C. (2007). The prevalence and correlates of eating disorders in the National Comorbidity Survey Replication. Biol. Psychiatry.

[48] Guerdjikova A.I., O’Melia A.M., Mori N., McCoy J., McElroy S.L. (2012). Binge eating disorder in elderly individuals. Int. J. Eat. Disord..

[49] Klump K.L., Racine S., Hildebrandt B., Sisk C.L. (2013). Sex differences in binge eating patterns in male and female adult rats. Int. J. Eat. Disord..

[50] Johnsen L.A., Gorin A., Stone A.A., le Grange D. (2003). Characteristics of binge eating among women in the community seeking treatment for binge eating or weight loss. Eat. Behav..

[51] Johnson W.G., Rohan K.J., Kirk A.A. (2002). Prevalence and correlates of binge eating in white and African American adolescents. Eat. Behav..

[52] Kinzl J.F., Traweger C., Trefalt E., Mangweth B., Biebl W. (1999). Binge eating disorder in females: A population-based investigation. Int. J. Eat. Disord..

[53] Houle-Johnson S.A., Kakinami L. (2018). Do sex differences in reported weight loss intentions and behaviours persist across demographic characteristics and weight status in youth? A systematic review. BMC Public Health.

[54] Serdula M.K., Collins M.E., Williamson D.F., Anda R.F., Pamuk E., Byers T.E. (1993). Weight control practices of US adolescents and adults. Ann. Intern. Med..

[55] Anversa R.G., Muthmainah M., Sketriene D., Gogos A., Sumithran P., Brown R.M. (2021). A review of sex differences in the mechanisms and drivers of overeating. Front. Neuroendocrinol..

[56] Lewinsohn P.M., Striegel-Moore R.H., Seeley J.R. (2000). Epidemiology and natural course of eating disorders in young women from adolescence to young adulthood. J. Am. Acad. Child Adolesc. Psychiatry.

[57] Stice E., Marti C.N., Rohde P. (2013). Prevalence, incidence, impairment, and course of the proposed DSM-5 eating disorder diagnoses in an 8-year prospective community study of young women. J. Abnorm. Psychol..

[58] Brown T.A., Forney K.J., Klein K.M., Grillot C., Keel P.K. (2020). A 30-year longitudinal study of body weight, dieting, and eating pathology across women and men from late adolescence to later midlife. J. Abnorm. Psychol..

[59] Mitchison D., Mond J. (2015). Epidemiology of eating disorders, eating disordered behaviour, and body image disturbance in males: A narrative review. J. Eat. Disord..

[60] Murray S.B., Nagata J.M., Griffiths S., Calzo J.P., Brown T.A., Mitchison D., Blashill A.J., Mond J.M. (2017). The enigma of male eating disorders: A critical review and synthesis. Clin. Psychol. Rev..

[61] Ridout S.J., Ridout K.K., Kole J., Fitzgerald K.L., Donaldson A.A., Alverson B. (2021). Comparison of eating disorder characteristics and depression comorbidity in adolescent males and females: An observational study. Psychiatry Res..

[62] Davis H.A., Graham A.K., Wildes J.E. (2020). Overview of binge eating disorder. Curr. Cardiovasc. Risk Rep..

[63] Wildes J.E., Forbush K.T. (2015). Ethnicity as a risk factor for eating disorders. The Wiley Handbook of Eating Disorders.

[64] Williams M.T., Lewthwaite H., Fraysse F., Gajewska A., Ignatavicius J., Ferrar K. (2021). Compliance with mobile ecological momentary assessment of self-reported health-related behaviors and psychological constructs in adults: Systematic review and meta-analysis. J. Med. Internet Res..

[65] Wrzus C., Neubauer A.B. (2023). Ecological momentary assessment: A meta-analysis on designs, samples, and compliance across research fields. Assessment.

[66] Forester G., Schaefer L.M., Dodd D.R., Burr E.K., Bartholomay J., Berner L.A., Crosby R.D., Peterson C.B., Crow S.J., Engel S.G. (2023). Time-of-day and day-of-week patterns of binge eating and relevant psychological vulnerabilities in binge-eating disorder. Int. J. Eat. Disord..

[67] Leehr E.J., Krohmer K., Schag K., Dresler T., Zipfel S., Giel K.E. (2015). Emotion regulation model in binge eating disorder and obesity-a systematic review. Neurosci. Biobehav. Rev..

[68] Svaldi J., Caffier D., Tuschen-Caffier B. (2010). Emotion suppression but not reappraisal increases desire to binge in women with binge eating disorder. Psychother. Psychosom..

[69] Dingemans A., Danner U., Parks M. (2017). Emotion regulation in binge eating disorder: A review. Nutrients.

[70] Coffino J.A., Udo T., Grilo C.M. (2019). The Significance of Overvaluation of Shape or Weight in Binge-Eating Disorder: Results from a National Sample of U.S. Adults. Obesity.

[71] Vartanian L.R., Porter A.M. (2016). Weight stigma and eating behavior: A review of the literature. Appetite.

[72] Rasmusson G., Lydecker J.A., Coffino J.A., White M.A., Grilo C.M. (2019). Household food insecurity is associated with binge-eating disorder and obesity. Int. J. Eat. Disord..

